# Nanoscale Synaptic Membrane Mimetic Allows Unbiased High Throughput Screen That Targets Binding Sites for Alzheimer’s-Associated Aβ Oligomers

**DOI:** 10.1371/journal.pone.0125263

**Published:** 2015-04-30

**Authors:** Kyle C. Wilcox, Matthew R. Marunde, Aditi Das, Pauline T. Velasco, Benjamin D. Kuhns, Michael T. Marty, Haoming Jiang, Chi-Hao Luan, Stephen G. Sligar, William L. Klein

**Affiliations:** 1 Department of Neurobiology, Northwestern University, Evanston, IL, United States of America; 2 Department of Comparative Biosciences, University of Illinois—Urbana Champaign, Urbana, IL, United States of America; 3 Department of Biochemistry, University of Illinois—Urbana Champaign, Urbana, IL, United States of America; 4 Department of Chemistry, University of Illinois—Urbana Champaign, Urbana, IL, United States of America; 5 High Throughput Analysis Laboratory and Department of Molecular Biosciences, Northwestern University, Evanston, IL, United States of America; Universitat Autònoma de Barcelona, SPAIN

## Abstract

Despite their value as sources of therapeutic drug targets, membrane proteomes are largely inaccessible to high-throughput screening (HTS) tools designed for soluble proteins. An important example comprises the membrane proteins that bind amyloid β oligomers (AβOs). AβOs are neurotoxic ligands thought to instigate the synapse damage that leads to Alzheimer’s dementia. At present, the identities of initial AβO binding sites are highly uncertain, largely because of extensive protein-protein interactions that occur following attachment of AβOs to surface membranes. Here, we show that AβO binding sites can be obtained in a state suitable for unbiased HTS by encapsulating the solubilized synaptic membrane proteome into nanoscale lipid bilayers (Nanodiscs). This method gives a soluble membrane protein library (SMPL)—a collection of individualized synaptic proteins in a soluble state. Proteins within SMPL Nanodiscs showed enzymatic and ligand binding activity consistent with conformational integrity. AβOs were found to bind SMPL Nanodiscs with high affinity and specificity, with binding dependent on intact synaptic membrane proteins, and selective for the higher molecular weight oligomers known to accumulate at synapses. Combining SMPL Nanodiscs with a mix-incubate-read chemiluminescence assay provided a solution-based HTS platform to discover antagonists of AβO binding. Screening a library of 2700 drug-like compounds and natural products yielded one compound that potently reduced AβO binding to SMPL Nanodiscs, synaptosomes, and synapses in nerve cell cultures. Although not a therapeutic candidate, this small molecule inhibitor of synaptic AβO binding will provide a useful experimental antagonist for future mechanistic studies of AβOs in Alzheimer’s model systems. Overall, results provide proof of concept for using SMPLs in high throughput screening for AβO binding antagonists, and illustrate in general how a SMPL Nanodisc system can facilitate drug discovery for membrane protein targets.

## Introduction

Membrane proteins mediate cell-cell communication events that provide important drug targets. High-throughput screening (HTS) that targets membrane proteins is showing progress [1–4], but membrane proteins are still largely inaccessible to biochemical HTS assays optimized for soluble protein targets. Although membrane proteins can be solubilized and purified from heterologous expression systems using detergents [5], their critical structural and functional integrity can be lost. A challenge is how to solubilize membrane proteins in a way that maintains protein structure and is adaptable to HTS platforms.

An appealing approach to preserving membrane protein integrity in a soluble state is to incorporate the proteins into Nanodiscs. Nanodiscs are self-assembling nanoscale phospholipid bilayers stabilized by engineered membrane scaffold proteins (MSP) [6–8]. Thus a membrane protein in a Nanodisc is soluble but nonetheless exists in a virtually native environment. To extend the utility of Nanodiscs beyond purified recombinant membrane proteins, we recently described a “Solubilized Membrane Protein Library” (SMPL) [9]. SMPL Nanodiscs can incorporate full membrane proteomes isolated directly from a biological tissue as a heterogeneous mixture of individualized proteins. Importantly, because of the affinity tags engineered on the MSP, it is possible to combine SMPL Nanodiscs with components of HTS assays designed for soluble proteins. This combination yields a platform for carrying out unbiased biochemical HTS assays of unknown targets derived from a specified membrane proteome.

As a first example of a Nanodisc-enabled HTS that targets an unknown membrane protein, we have applied SMPL Nanodiscs to target the neuronal binding of Aβ oligomers (AβOs). AβOs are assemblies of cell-secreted Aβ peptides whose ligand activity is implicated in Alzheimer’s disease (AD) pathogenesis [10,11]. AβOs associate with a binding site present at the synaptic membrane, but its identity is the subject of many divergent hypotheses [12–21]. While AβO binding can be assessed using intact neurons, exposure to AβOs induces receptor clustering [17,22] and internalization [12,20,23,24], suggesting that complex mechanisms regulate the distribution of bound AβOs. To facilitate receptor discovery efforts, and establish an HTS strategy to identify molecules that prevent synaptic AβO binding, an assay that removes the event of AβO binding from its cellular context is called for.

Here we establish a new paradigm for AβO binding assays using SMPL Nanodiscs incorporating the synaptic membrane proteome. We implement this AβO binding system in a first-of-its-kind unbiased HTS assay for AβO binding antagonists. One small molecule identified through our Nanodisc-based HTS shows potent inhibition of synaptic AβO binding.

## Results

### Identification of synaptic AβO receptors is complicated by detergent-resistant associations with other synaptic membrane proteins

**Fig 1:**
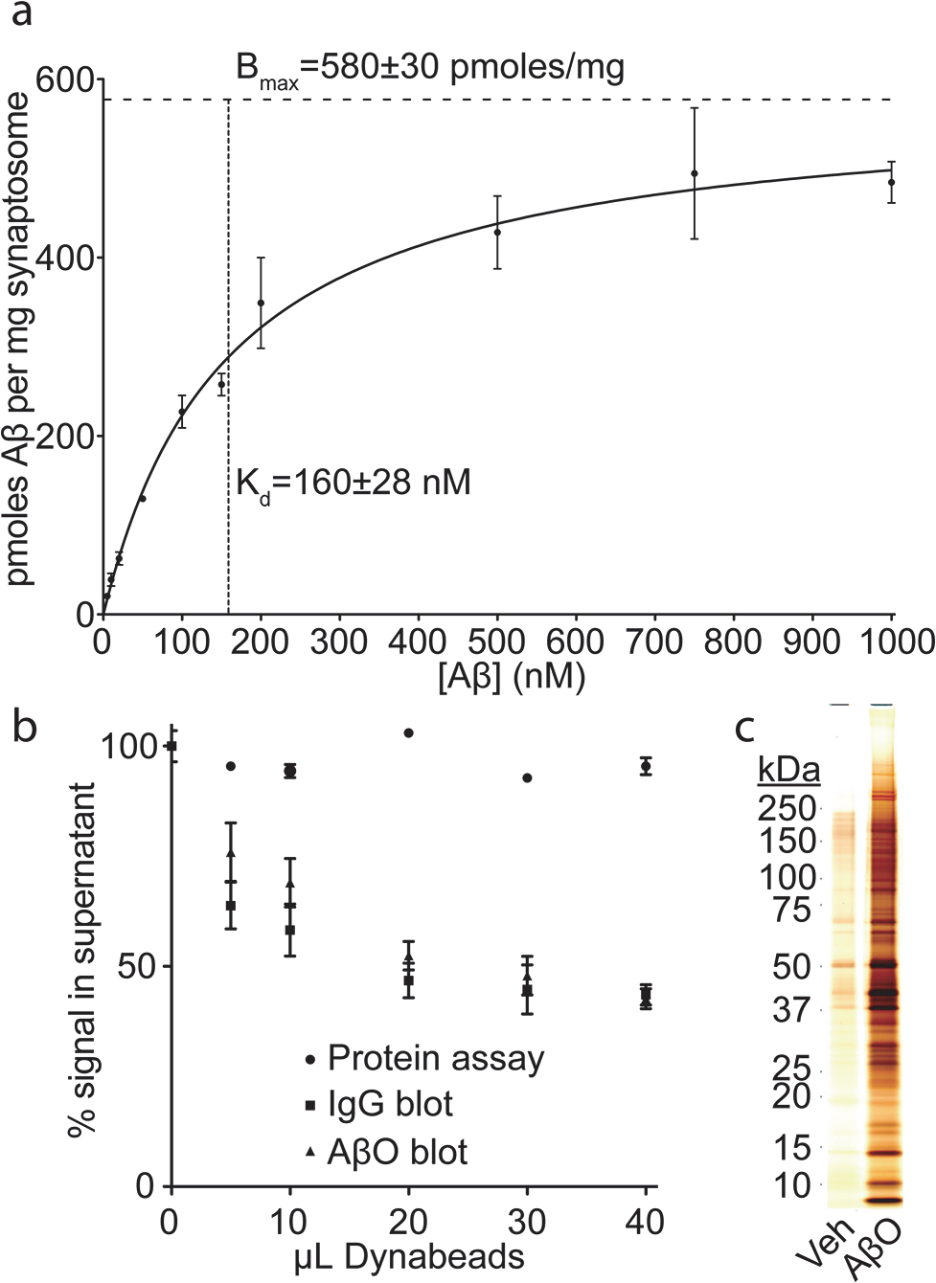
AβO receptors are highly connected. Rat cortical synaptosomes were titrated in solution with AβOs and binding was detected by dot immunoblot (Mean +/- SD; n = 3) (a). AβO-bound synaptosomes were broken down with triton and deoxycholate to maintain AβO interactions with the binding site and detergent-lysates were immunodepleted using AβO-specific NU2 antibody. A negligible fraction of total synaptosomal protein is co-immunoprecipitated, illustrating a highly selective binding (Mean +/- SD; n = 2) (b). The co-immunoprecipitated fraction was analyzed by SDS-PAGE and silver staining to assess the number and size of proteins associated with an AβO binding complex (c).

The motivation to create a soluble synaptic membrane mimetic stems from the difficulty in identifying unique membrane binding sites for AβOs. To verify the involvement of receptors in AβO binding, we employed synaptosomes—an established model for synaptic biology previously used in AβO studies [17,23]. Using a centrifugation assay coupled to dot immunoblots, we found that AβO binding is saturable with high affinity (K_d_ = 160±28 nM, Aβ monomer equivalent; B_max_ = 580±30 pmoles Aβ mg^−1^ synaptosomes) (Fig 1A). Next, we attempted to isolate synaptosomal AβO binding proteins through co-immunoprecipitation. Synaptosomes were incubated with AβOs, treated with NU2 oligomer-sensitive antibody, and solubilized with detergents. Pull down using anti-mouse IgG magnetic beads (Fig 1B) provided a fraction that, when analyzed by SDS-gels and silver stain, showed a broad array of synaptosomal proteins (Fig 1C). Despite this co-immunoprecipitation of a large number of proteins, there was virtually no reduction of synaptosomal protein from the detergent lysates (Fig 1B), indicating the AβO binding sites constitute a tiny fraction of total synaptosomal protein. We concluded that AβOs bind to a small number of specific sites within a detergent-resistant lateral membrane protein complex.

### Solubilized synaptic membranes are captured in SMPL Nanodiscs while retaining native protein composition and function

**Fig 2:**
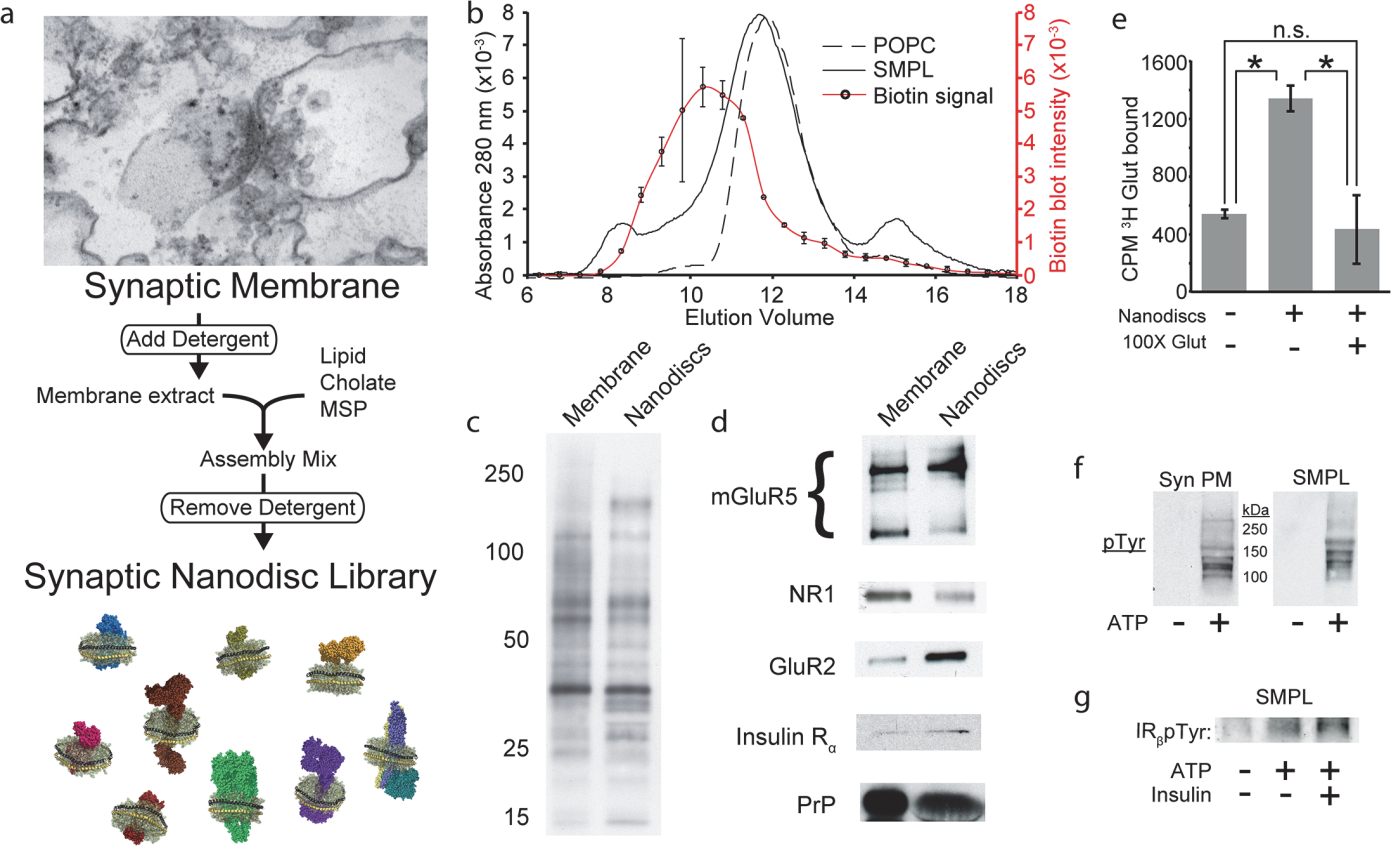
Nanodiscs preserve synaptic protein composition and structure. (a) Schematic of SMPL Nanodisc formation using synaptic plasma membranes. (b) Nanodiscs containing biotinylated synaptic membranes (solid curve) or POPC (dashed curve) were separated by size exclusion chromatography. Fractions eluting from the synaptic Nanodisc run were collected and analyzed by dot blot to locate biotinylated synaptic proteins (red curve; Mean +/-SD; n = 2). The population of biotinylated membrane proteins inserted into Nanodiscs was analyzed by SDS-PAGE, probing for biotin (c) or using antibodies against specific proteins related to AβO binding (d). ^3^H glutamate binding to SMPL Nanodiscs was assessed in the absence and presence of a 100-fold excess of cold glutamate (Mean +/- SD; n = 3; * p<0.05) (e). Enzymatic activity was assessed in synaptic plasma membranes (Syn PM) and SMPL Nanodiscs (SMPL) by probing for tyrosine phosphorylation in the absence and presence of ATP (f). Insulin receptor activity was probed using an antibody recognizing IR_β_pTyr^1162/1163^ (g).

We used SMPL Nanodiscs [9] to divide large synaptic membrane protein networks into small units for use in AβO binding studies (Fig 2A). To track the incorporation of tissue-derived proteins into Nanodiscs, we biotinylated synaptic membranes prior to Nanodisc formation. Biotin quantification in size exclusion column fractions revealed a protein-containing subpopulation of Nanodiscs whose mean Stokes diameter is increased by 3.3 nm relative to Nanodiscs containing only palmitoyl-oleoyl-phosphatidylcholine (POPC), which constitute a majority of Nanodiscs in the SMPL population (Fig 2B). This difference likely reflects the presence of membrane protein domains that extend in a direction normal to the plane of the Nanodisc lipid bilayer rather than an increase in disc diameter. The majority of the biotin-tagged synaptic surface proteins are present in Nanodiscs (Fig 2C), including diverse classes of membrane proteins verified by Western blot (Fig 2D): G protein coupled receptors (e.g. mGluR5), subunits of AMPA- and NMDA-type glutamate receptors (GluR2, NR1), receptor tyrosine kinases (e.g. insulin receptor) and membrane-associated proteins (cellular prion protein, PrP^C^).

We evaluated the functional status of synaptic membrane proteins in Nanodiscs using ligand binding and enzymatic activity as a proxy for correct protein folding. SMPL Nanodiscs were incubated with ^3^H glutamate alone or with a 100-fold excess of cold glutamate (Fig 2E). Mimicking intact membrane behavior (Supporting Information S1 Fig), Nanodiscs containing synaptic membrane proteins bind glutamate, and competition by excess cold glutamate reduced the signal to background levels. A 60% reduction in glutamate binding to Nanodiscs incorporating trypsinized synaptic membranes indicates a requirement for intact glutamate receptors (Supporting Information S1 Fig). To further test whether Nanodisc-encapsulated proteins retain their proper structure, we assayed tyrosine kinase activity (Fig 2F andg). ATP stimulates tyrosine phosphorylation of multiple substrates in SMPL Nanodiscs, including at tyrosine 1162/1163 in the insulin receptor β-subunit. While it is impossible to assay each receptor in SMPL Nanodiscs for structure and activity, these data suggest that multiple classes of synaptic proteins assume their native structures in Nanodiscs and are consistent with published reports of functional protein incorporation in Nanodiscs [25–27].

### AβOs bind SMPL Nanodiscs at a protease sensitive binding site

**Fig 3:**
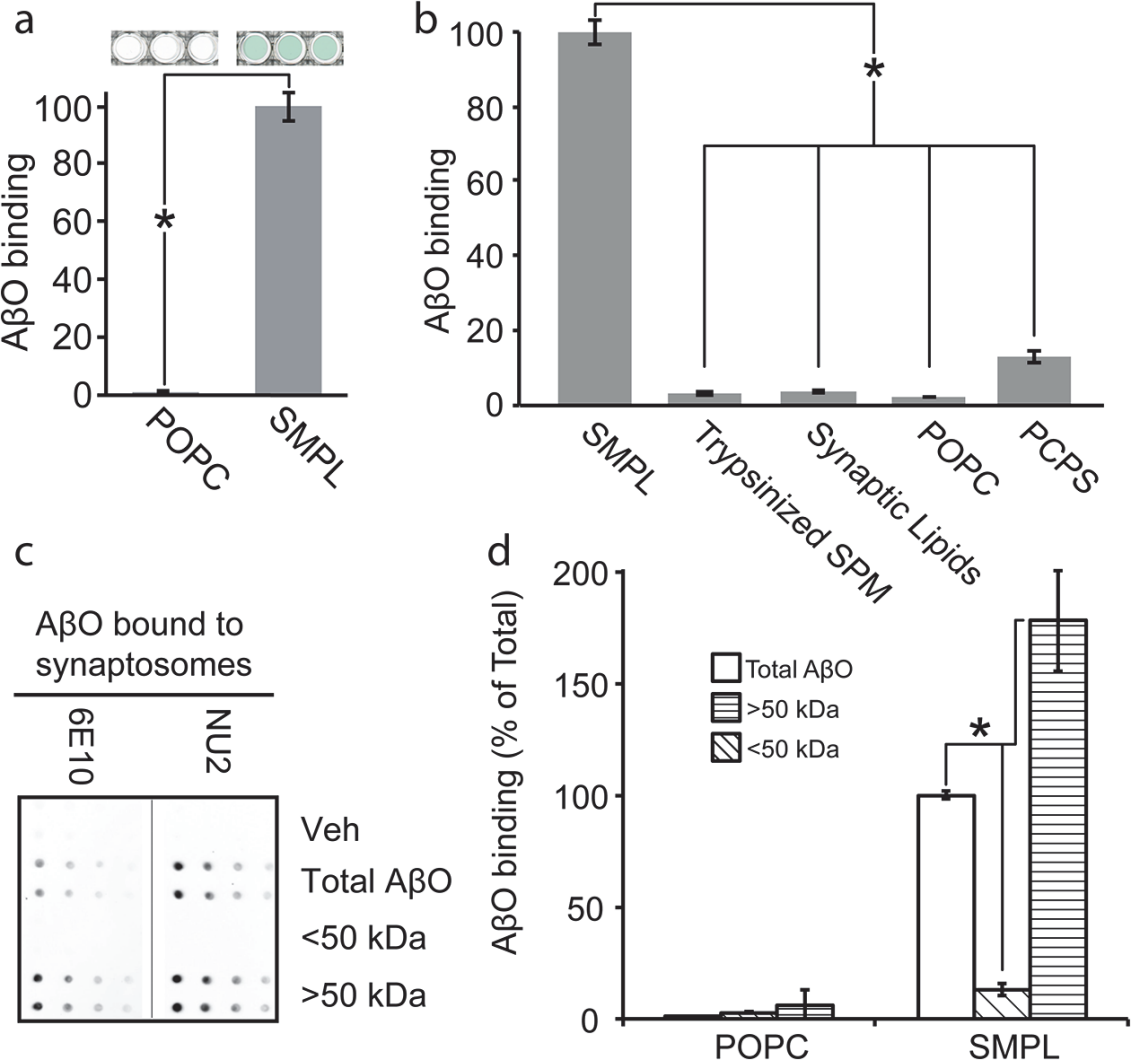
Synaptic Nanodiscs contain an AβO binding protein that interacts selectively with high molecular weight oligomers. A Nanodisc-based assay for AβO binding indicates the transfer of the AβO binding site into Nanodiscs (a) (n = 3; mean +/- SD; * p<0.05). Nanodiscs containing a range of detergent-solubilized precursor membranes or lipid mixtures were applied to the AβO binding assay (b; SPM—synaptic plasma membrane, Trypsinized SPM—SPM treated with trypsin prior to Nanodisc incorporation, Synaptic Lipids—lipids extracted from SPM, POPC—100% POPC lipid, PC/PS—1:1 POPC:POPS; n = 3; mean +/- SD; * p<0.05). AβOs were separated into populations smaller (diagonal fill) and larger (horizontal fill) than 50 kDa and assayed for binding to synaptosomes (c) and Nanodiscs (d). (n = 2; mean +/- SD; * p<0.05)

We hypothesized that the synaptic proteome contains a synaptic surface protein responsible for mediating AβO binding and predicted that its inclusion in a Nanodisc library should produce oligomer-binding properties similar to that of the source membranes. Using the hexahistidine tags present on every MSP, we immobilized synaptic SMPL Nanodiscs on magnetic beads and exposed them to AβOs. We probed for bound AβOs using an antibody-based colorimetric assay. SMPL Nanodiscs produced a strong binding response compared to bare POPC Nanodiscs (Fig 3A). We concluded that the Nanodiscs incorporate AβO binding sites from the synaptic plasma membrane.

While our hypothesis that AβO binding occurs to a specific, proteinaceous receptor is substantiated by a large body of evidence [12–24,28,29], another prominent hypothesis states that AβOs interact directly with lipid bilayers [30–33]. To distinguish between protein and lipid AβO binding sites, we created a panel of Nanodiscs containing a variety of membrane extracts or artificial lipid mixtures (Fig 3B). Removal of proteins from the source membranes through trypsinization or protein precipitation to yield a synaptic lipid extract eliminated oligomer binding to SMPL Nanodiscs. Introducing equimolar palmitoyl-oleoyl-phosphatidylserine (POPS) into neutral POPC Nanodiscs elicited weak AβO binding. This response is likely due to nonspecific interactions with the high negative charge of phosphatidylserine, whose natural abundance in synaptic plasma membrane is only 12% [34]. These results support the hypothesis that AβO binding occurs via an association with a membrane protein.

### Nanodiscs retain cell-like selectivity for binding high MW AβOs

Based on previous findings that attribute AβO binding and synaptotoxicity to a species with a molecular weight larger than 50 kDa [17,23,35], we tested whether AβO receptors in synaptosomes would preferentially associate with high molecular weight AβOs. We fractionated AβOs into species smaller and larger than 50 kDa and assayed for binding to synaptosomes. Bound AβOs were detected in a dot immunoblot procedure using NU2 oligomer-specific antibody or 6E10 Aβ-specific antibody (Fig 3C). Binding-competent species are present in unfractionated AβOs and AβOs larger than 50 kDa but not in the fraction smaller than 50 kDa.

SMPL Nanodiscs show selectivity for high MW AβOs similar to the selectivity observed in cells and synaptosomes. We compared unfractionated, high MW, and low MW AβOs using our SMPL-based AβO binding assay (Fig 3D). We used biotin-labeled AβOs to avoid preferential antibody detection of specific Aβ species. High MW oligomers produce an 80% increase in assay response relative to unfractionated AβOs. Low MW oligomers do not show an appreciable binding signal. These results establish that SMPL Nanodiscs contain a binding site that discriminates among AβO species—interacting with an oligomeric species larger than 50 kDa (12-mer or greater).

### AβO binding shows receptor-ligand behavior

**Fig 4:**
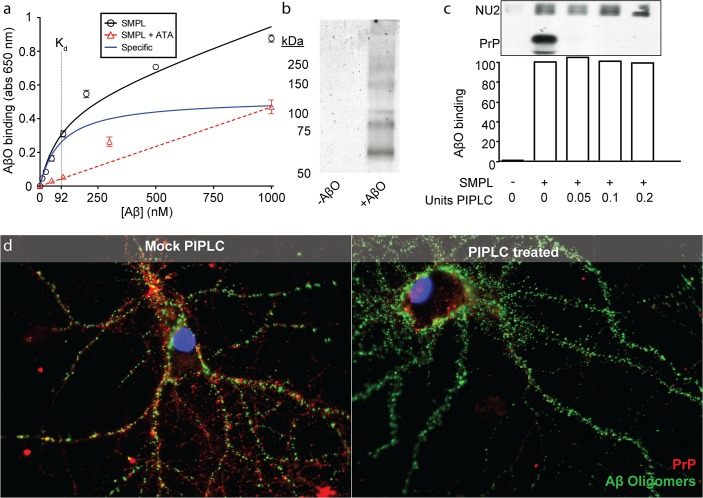
AβO binding is receptor-mediated but PrP^C^-independent in Nanodiscs and mature neurons. Immobilized SMPL Nanodiscs were titrated with AβOs and bound AβOs were detected with NU2 oligomer-specific antibody coupled to an HRP-based colorimetric assay. Nonspecific binding was measured in the presence of excess ATA, which blocks AβO/receptor binding, and used to calculate the specific binding component (blue). n = 3; mean +/- SD. (a). To analyze Nanodisc proteins co-immunoprecipitating with AβOs, Nanodiscs containing biotinylated synaptic plasma membranes were affinity precipitated and visualized by SDS-PAGE immunoblot with biotin detection (b). To test the prediction that PrP^C^ mediates AβO binding, immobilized Nanodiscs were split into four equivalent reactions and pre-treated with 0, 0.05, 0.1, or 0.2 units of PIPLC to remove PrP^C^ before exposing to AβOs and probing with NU2 as in (a). PrP^C^ removal was verified by Western blotting after the colorimetric assay was complete. NU2 bound to each immobilized Nanodisc/AβO complex is detected in the blot by anti-mouse secondary antibodies used to probe for the mouse antibody against PrP^C^ (c). The effect of PrP^C^ removal on AβO binding was tested using mature hippocampal cultures treated with PIPLC (d). PrP^C^ was detected using an antibody (red) and fluorescence-conjugated AβOs were visualized directly (green).

Oligomers bind with receptor-like saturation in neuronal cultures (K_d_ ≅ 100 nM, monomer equivalent) [17]. We exposed SMPL Nanodiscs to a range of AβO concentrations to assess the saturability of the binding site (Fig 4A). AβOs self-associate at high concentrations [36], which prohibits the use of excess unlabeled AβOs to measure nonspecific binding. We therefore used the AβO binding antagonist, aurin tricarboxylic acid (ATA; see below), to allow the determination of the specific AβO binding curve to Nanodiscs. In the presence of excess ATA, AβO binding shows linear dose dependence consistent with a nonspecific interaction. Specific AβO binding to SMPL Nanodiscs saturates with a K_d_ of 92 nM (Aβ monomer equivalent), consistent with our other estimates in hippocampal culture and cortical synaptosomes. We note that nonspecific AβO binding becomes dominant at Aβ concentrations greater than 1 μM.

Saturable AβO binding to SMPL Nanodiscs is consistent with specific oligomer binding to particular synaptic proteins. To analyze the proteins in SMPL Nanodiscs that interact with AβOs, we performed affinity pull-downs using antibody-immobilized AβOs and SMPL Nanodiscs that were biotinylated in order to enhance protein detection. In this analytical study, we collected six Nanodisc-encapsulated proteins ranging from 50 to >250 kDa (Fig 4B). Each of these bands represents a potential AβO-interacting protein, but also may represent receptor-interacting proteins or non binding-relevant elements of a multi-subunit AβO receptor. The selectivity of the SMPL-based pull-down provides a major improvement relative to the pull down from synaptosomes in Fig 1C and illustrates the general utility of SMPL Nanodisc immunoprecipitation to discover membrane protein binding partners.

### Initial binding of AβOs can occur in the absence of PrP^C^

The identity of neuronal membrane proteins that act as receptors for AβOs and mediate their synaptotoxicity is of great interest [10,37,38]. A notable candidate is the cellular prion protein, PrP^C^ [16]. While PrP^C^ has garnered support as a receptor [22,29,39], contradictory evidence exists in the literature [40–42]. Because Nanodiscs offer a new system for studying AβO binding in an isolated environment, we sought to investigate whether PrP^C^ acts to mediate AβO binding in the Nanodisc-based AβO binding assay. To test whether PrP^C^ is required for AβO binding in our simplified system, we treated SMPL Nanodiscs with phosphatidylinositol-specific phospholipase C (PIPLC) to remove the lipid anchor from PrP^C^. We found no loss of AβO binding (Fig 4C). Control Western blots of Nanodiscs following PIPLC treatment showed complete removal of PrP^C^ despite retention of binding signal. To substantiate this finding in a cellular system and test the similarity between neuron and Nanodisc binding in the context of receptor manipulations, we treated mature rat hippocampal neuron cultures with PIPLC. This caused a near-complete loss of PrP^C^ immunofluorescence in neuronal processes without reducing AβO binding (Fig 4D). Thus, PrP^C^ elimination does not eliminate AβO binding.

### HTS identification of aurin tricarboxylic acid as an AβO binding antagonist

An HTS assay monitoring AβO binding would enable efficient identification of binding antagonists from chemical compound libraries. A biochemical assay is desirable, particularly one that does not require prior knowledge of the receptor. While available assays measuring AβO binding to intact primary neuronal cultures are unbiased with respect to the receptor, they are subject to cell fluctuations and are microscopy-driven with a heavy data processing burden. SMPL Nanodiscs combine positive aspects of each approach into a single assay: Like cells, SMPL Nanodiscs contain a collection of synaptic membrane proteins including a receptor for AβO; like purified proteins in biochemical assays, Nanodiscs are soluble, scalable preparations amenable to long-term storage and adaptable to numerous detection modalities.

**Fig 5:**
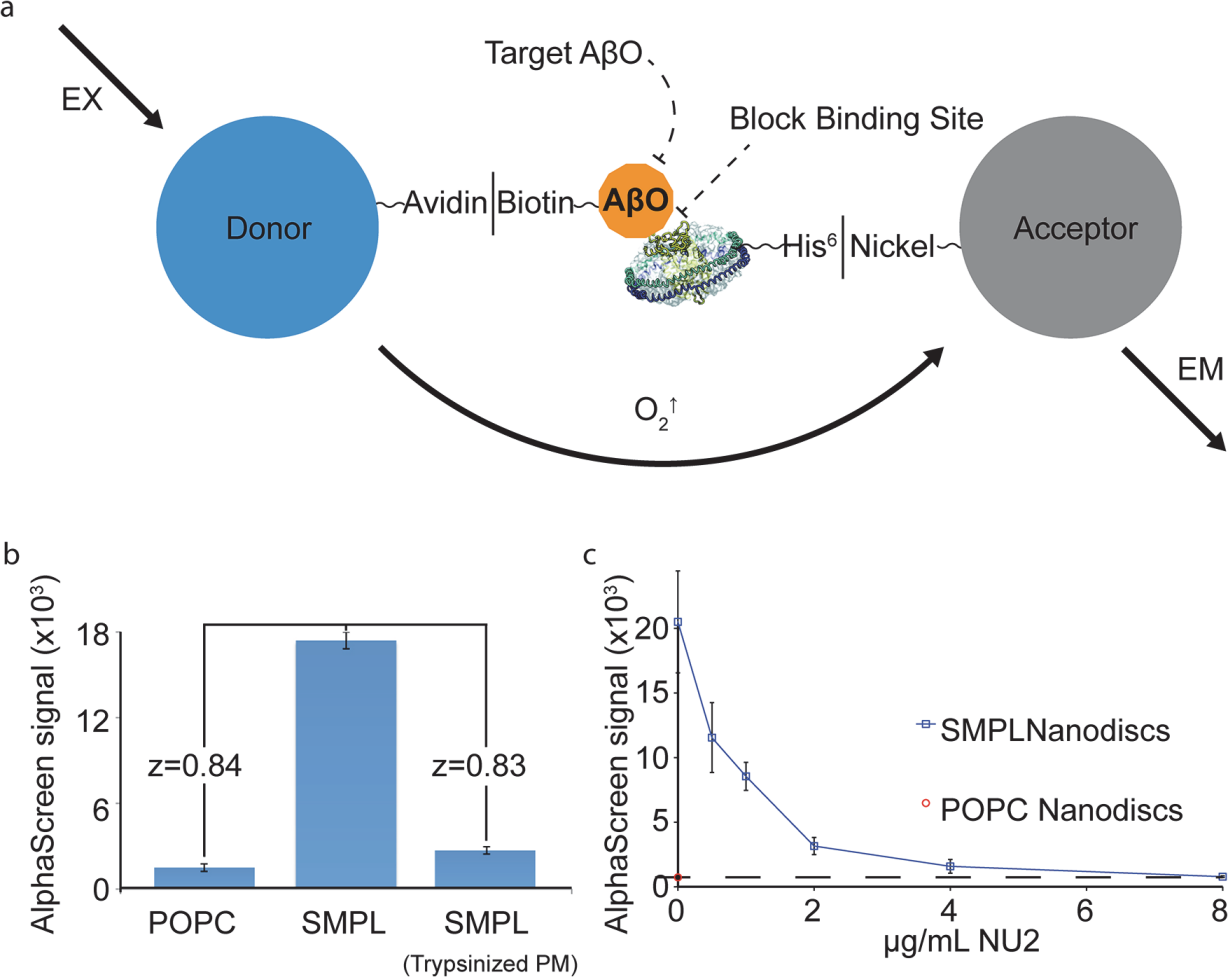
SMPL Nanodiscs provide the basis for a high-throughput assay for AβO binding antagonists. A schematic of the AlphaScreen assay adapted to measure AβO binding to synaptic Nanodiscs (a). Biotinylated AβOs and His-tagged MSP molecules link Nanodiscs to AlphaScreen donor and acceptor beads. The proof-of-concept assay produces high dynamic range (b). NU2 oligomer-specific antibodies were used as a drug stand-in to test the assay’s response to an applied treatment (c).

To establish an HTS assay for antagonists of AβO binding, we combined our system of synaptic membrane Nanodiscs and AβOs with the AlphaScreen assay. AlphaScreen is a homogeneous proximity assay consisting of chemiluminescent “acceptor” beads that produce light when brought together with photosensitive “donor” beads [43]. Nanodiscs are linked via hexahistidine tags to acceptor beads. Biotinylated AβOs link to streptavidin on donor beads. AβO/SMPL binding brings the beads into proximity to produce a chemiluminescent signal (Fig 5A). The AlphaScreen assay produces a strong AβO binding response that is sensitive to trypsinization of the source membranes (Fig 5B).

This assay can identify hit compounds that act through altering or concealing binding-related epitopes on either the receptor or AβOs. To test an oligomer-directed mechanism, we applied AβO-specific antibodies and demonstrated that the assay was sensitive to subtle decrements produced by an applied treatment, reducing the signal to the level of POPC Nanodiscs (Fig 5C). To address a receptor-targeted mechanism, we utilized soybean trypsin inhibitor (STI)—a protein we determined to bind to neurons in culture and reduce AβO binding by 87% (Supporting Information S2 Fig). We applied STI prior to AβOs in the AlphaScreen assay, where it reduced AβO binding signal by 67% at 1 mg mL^-1^ (Supporting Information S2 Fig).

**Fig 6:**
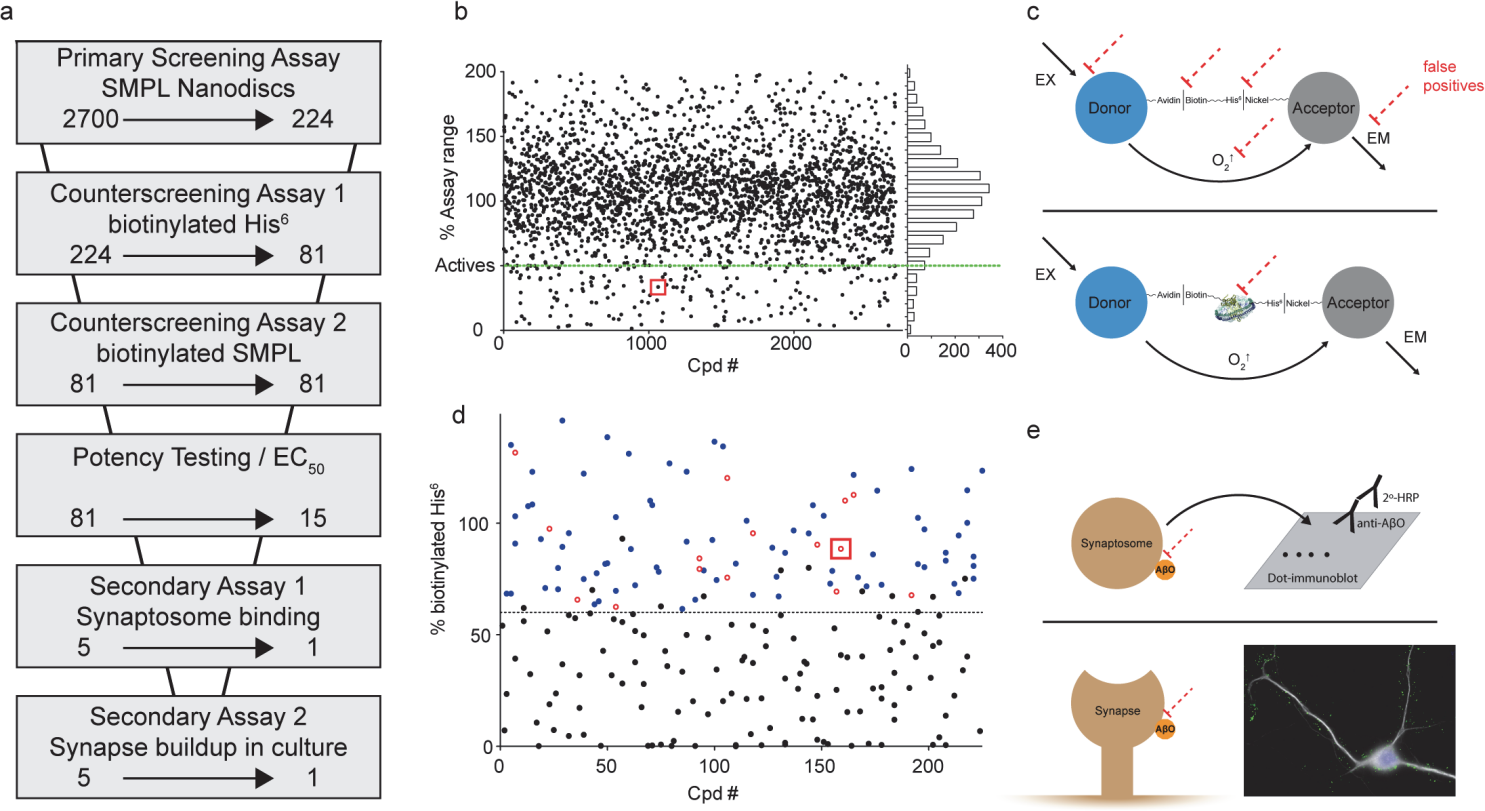
Screening strategy effectively eliminates false positives. (a) Screening assays used to evaluate the effect of Spectrum Collection molecules on AβO binding. The arrows indicate the reduction in compounds resulting at each step. (b) Compiled data from the primary AlphaScreen assay are shown in a single graph normalized to POPC and SMPL in-plate controls. (c) Schematics of counterscreening assays designed to identify false positive compounds acting on off-target elements of the primary screening assay (dashed red lines). Assays use AlphaScreen donor and acceptor beads linked together by either biotinylated hexahistidine (top) or Nanodiscs containing biotinylated synaptic proteins (bottom). (d) Data from the biotinylated hexahistidine counterscreen. Black symbols denote compounds classified as likely false positives. Blue symbols denote compounds that were retested in dose-response format (Examples shown in Fig 7), and the compounds showing significant signal reduction at 1 μM are shown as open red circles. (e) Secondary, orthogonal assays to verify compound efficacy in preventing AβO binding include a dot immunoblot test for AβO binding to rat cortical synaptosomes (Top; shown in Fig 8) and an immunocytochemical analysis of AβO binding to cultures of rat hippocampal neurons (Bottom; shown in Fig 9 for ATA). Red squares in panels b and d identify the data points associated with ATA.

To provide proof of principle for a large-scale screen for AβO binding antagonists, we tested 2700 small molecules and natural products from the Spectrum Collection. Our approach consisted of multiple phases, summarized in Fig 6A. As a primary assay, AlphaScreen was used to identify compounds that produced at least a 50% signal reduction defined by in-plate SMPL and POPC controls (Fig 6B). This criterion included 224 compounds—8.3% of the library. Well-well correlation between duplicates was good, with an overall R^2^ value of 0.78 (Supporting Information S3 Fig). The z factors [44] calculated between POPC and SMPL Nanodisc controls average 0.59 among the 17 plates used for the screen (Supporting Information S3 Fig).

Dual counterscreening assays were conducted on the 224 actives to eliminate from further consideration any assay-specific false positives (Fig 6C). The first counterscreening assay consisted of AlphaScreen donor and acceptor beads conjugated directly using biotinylated hexahistidine. This assay was designed to reveal any compounds that interrupt the biotin/streptavidin or nickel/His-tag connections in the AlphaScreen assay, as well as those compounds reducing the stimulation or detection of singlet oxygen molecules. This process produced 81 compounds with minimal false positive activity.

Another source of potential false positives is the disruption of Nanodisc integrity in such a way that reduces their ability to stabilize synaptic membrane proteins. To test for compounds that disrupt Nanodisc assembly, we used SMPL Nanodiscs containing biotinylated synaptic membrane proteins. When AlphaScreen donor and acceptor beads were directly conjugated via the biotinylated membrane SMPL, robust signal was achieved that was lost upon Nanodisc disassembly by cholate and SDS. However, none of the molecules tested in this assay showed a propensity to disrupt Nanodiscs.

**Fig 7:**
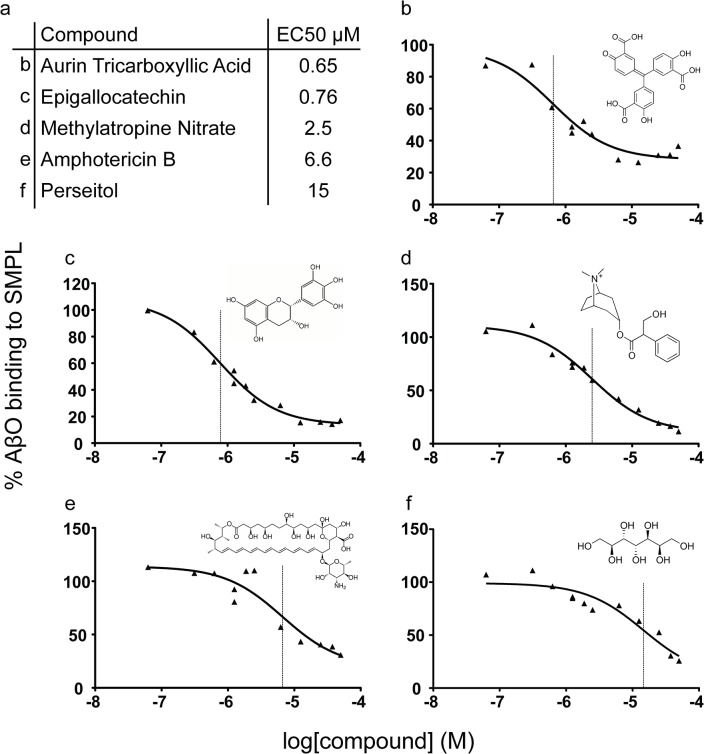
Dose response testing of selected compounds. Of fifteen compounds surviving dose-response potency testing, five commercially-available molecules were repurchased for further analysis. (a) The names and EC_50_ values of each are listed with the corresponding panel identifier. (b-f) Individual dose response curves and chemical structures corresponding to compounds listed in panel a. Vertical lines mark the EC_50_ values in each plot.

**Fig 8:**
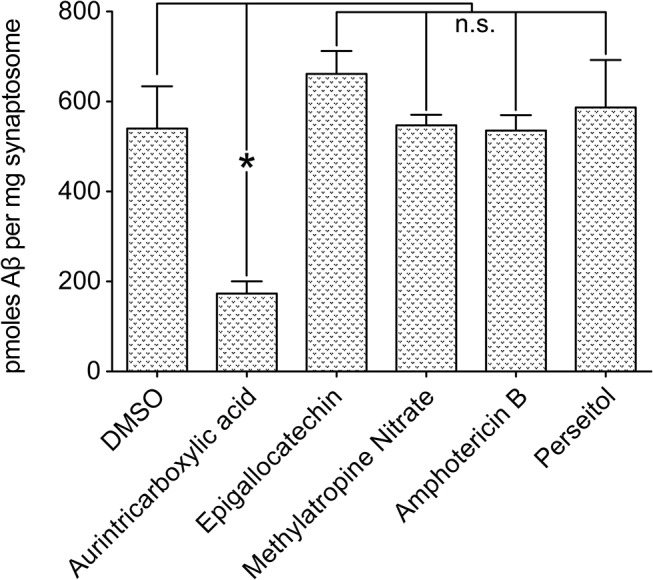
Impact of selected compounds on synaptosome binding. Selected compounds were repurchased and tested for an impact on AβO binding to rat cortical synaptosomes in a dot immunoblot assay. n = 3; Mean+/-SEM.

The 81 Compounds remaining after the biotinylated hexahistidine counterscreen were re-screened in a 6-point dose-response format using the primary AlphaScreen assay. We identified 15 compounds that produced sustained inhibition at 1.25 μM and performed expanded dose response testing to measure the EC_50_. Of the 15 compounds, five (Fig 7) were repurchased based on commercial availability and tested in an orthogonal screening assay measuring the binding of AβOs to synaptosomes (Fig 8). Aurin tricarboxylic acid (ATA) was the only compound that produced a reduction in synaptosome binding.

**Fig 9:**
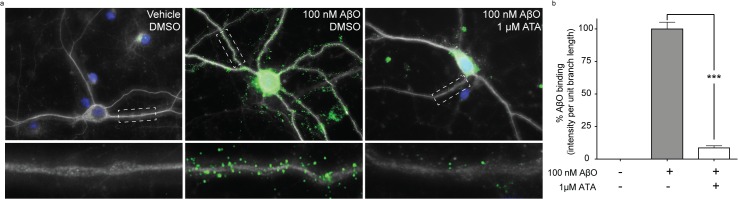
Aurin tricarboxylic acid potently reduces synaptic AβO accumulation in culture. Aurin tricarboxylic acid was assayed at 1μM for a preventative effect on AβO accumulation at synapses in cultured rat hippocampal neurons. (a) Typical neurons after treatment with Vehicle (left), AβOs (center), or AβOs following ATA pre-treatment (right). AβOs are shown in green, neurons identified by β3 tubulin fluorescence are white, and DAPI is blue to indicate nuclei. Selected neurites are enlarged below each image to illustrate the distribution of bound AβOs. (b) Quantification of AβO intensity per neurite branch length as a percent of AβO treated neurons. *** Denotes p<0.0001.

We tested the effect of ATA pre-incubation on the synaptic accumulation of AβOs. ATA was applied at 1 μM to mature cultures of rat hippocampal neurons for 30 minutes before the addition of 100 nM AβOs. ATA pre-treatment reduced AβO binding along neurite branches by 91% (Fig 9).

These results establish a reliable HTS approach using SMPL Nanodiscs in various AlphaScreen assay formats to efficiently screen small molecule libraries and derive meaningful hit compounds.

## Discussion

We have developed a nanobiotechnology that allows a new approach to HTS for membrane protein targets. It is based on a procedure that solubilizes an entire membrane proteome into an artificial lipid bilayer environment favoring physiological membrane protein function. We have applied it to proteins extracted from the plasma membrane of central nervous system synapses to obtain an unbiased population of solubilized synaptic membrane proteins that are enzymatically active and capable of binding physiological and pathological ligands. The usefulness of the method is illustrated by a novel HTS assay designed to discover compounds that block Alzheimer’s-related toxins from binding membrane protein targets. Through a stepwise process of primary screening, counterscreening, potency determinations, and secondary screening techniques, we eliminated irrelevant compounds from consideration and identified a single antagonist of synaptic AβO accumulation. These results provide proof of concept that small organic molecules can block interactions between a protein toxin and its membrane protein target. The technology described is readily adaptable to HTS of other protein-membrane protein interactions.

Our approach relies on the unique properties of Nanodiscs to provide a solubilized membrane protein library (SMPL) [9] that includes potential AβO receptors in a state that is ideal for HTS. SMPL Nanodiscs combine positive aspects of cell-based and biochemical (pure-protein) methods into a single assay: like cells, SMPL Nanodiscs contain a collection of synaptic membrane proteins including receptors for AβOs; like purified proteins in biochemical assays, Nanodiscs are soluble, readily scalable preparations amenable to long-term storage. Affinity tags engineered onto every MSP molecule allow SMPL Nanodiscs to readily apply HTS to entire membrane proteomes. For unknown targets, the same material used in unbiased assays can be used for receptor discovery, as illustrated by improvements over synaptosome-based co-immunoprecipitation techniques described above.

Nanodiscs that separate membrane proteomes into usable units provide an improved means for studying the synaptic plasma membrane, which contains disease-relevant targets in a complex lateral network. By distributing the synaptic membrane proteome into Nanodiscs, we create a powerful new tool to probe synaptic targets. We show that SMPL Nanodiscs effectively model the diverse content of the synaptic membrane proteome and maintain them in a functional state and serve as a proof of concept for other membrane systems beyond the current application.

Synaptic SMPL Nanodiscs are well suited to address the binding of Alzheimer’s-associated toxins. AβOs bind to a mobile site that becomes immobilized at synapses along with a variety of synaptic proteins that mount a toxic signaling response [17]. When the molecular components of the synaptic membrane are divided into Nanodiscs, binding occurs in isolation from secondary clustering or organizing mechanisms. In this system, the protein and lipid components can be strictly controlled, allowing us to directly address the hypothesis that lipids mediate Aβ peptide binding [30,31]. Through a series of experiments examining the molecular nature of the AβO binding site, we observed that binding to Nanodiscs requires the protein component of synaptic plasma membranes. Lipids alone were insufficient, though superphysiological amounts of phosphatidylserine produced a small increase in AβO binding consistent with reports of Aβ peptide binding through an electrostatic mechanism [45]. While we did not specifically test the impact of GM1 gangliosides [46,47] on AβO binding in our system, we note that trypsin treatment, while expected to spare gangliosides [48,49], eliminates AβO binding when treated membranes are incorporated into Nanodiscs (Fig 3B). AβO binding to SMPL Nanodiscs was saturable with high affinity and showed a cell-like selectivity among AβO subspecies, favoring AβOs larger than 50 kDa [17,23,35]. These findings support a model in which AβOs bind a specific synaptic protein with receptor-like characteristics.

Because of the interest and controversy surrounding PrP^C^ as a binding site for AβOs, we used our system to investigate its involvement under the conditions of our SMPL Nanodisc-based assay. We found that PIPLC removes PrP^C^ from SMPL Nanodiscs and mature neuronal cultures without perturbing AβO binding, contradicting a model where PrP^C^ acts as a direct receptor for AβOs [16]. This finding does not rule out an indirect role for PrP^C^ in AβO attachment, but the similar response of a SMPL-based and neuron-based assay following modulation of an AβO receptor candidate demonstrates the fidelity of synaptic SMPL Nanodiscs for AβO binding and reveals that our HTS assay monitors AβO binding to receptors other than PrP^C^,

A SMPL Nanodisc system allows assays monitoring AβO binding sites derived from intracellular membranes as well as the cell surface. While extracellular AβOs accumulate in an age-dependent fashion in AD transgenic mice [50], intraneuronal Aβ is also observed in AD mouse models [51–53]. The relative contributions of these pools of AβOs to memory loss are unclear. Recently, intranasal injection of 5xFAD mice with an AβO-specific antibody improved memory suggesting the presence of memory-relevant AβO species in the extracellular space [54]. MRI probes conjugated to these AβO-specific antibodies reveal the accumulation of surface-associated AβO in living animals [55], showing that surface AβOs have potential diagnostic as well as therapeutic value. An antagonist of AβO binding that reaches the interstitial space of the brain would therefore be positioned correctly to act against memory-relevant AβO species with a non-invasive biomarker to evaluate efficacy.

Solution-based biochemical methods have previously been devised for screening antagonists of protein-protein interactions (PPI) [43], and these interactions have been successfully targeted by small molecules [56]. Our approach brings solution-based assays to bear on PPI involving membrane proteins, including unidentified receptors for soluble protein ligands. Here, SMPL Nanodiscs containing synaptic membrane proteins were used to discover small molecules that block AβO binding. Because AβOs must maintain a binding-competent conformation to produce signal in our assay, the approach is capable of identifying modifiers of AβO binding propensity as well as blockers of AβO/receptor binding.

The application of synaptic SMPL Nanodiscs to Alzheimer’s high throughput screening serves as a proof of concept for similar screens using other membrane mimetic SMPL Nanodiscs. We envision that similar preparations will be adaptable to drug discovery, target identification, and interactomics (e.g. investigations of cell adhesion molecules or mediators of vesicle trafficking). In these applications, system-specific testing must be carried out on a case-by-case basis as we have done for AβOs to show fidelity to intact membrane systems.

Synaptic SMPL Nanodiscs have been useful in answering specific questions about the nature of AβO binding (e.g. testing protein- versus lipid-based binding) and at the same time have provided a powerful unbiased system to screen for antagonists without knowing the receptor. A rapid screening of 2700 small molecules identified ATA as a potent antagonist of AβO binding. ATA was effective at reducing binding to SMPL Nanodiscs, cortical synaptosomes, and intact synapses in hippocampal neuron cultures. As a binding antagonist, ATA provides a useful experimental tool, for example in determining the K_d_ for specific AβO binding (Fig 4A). More importantly, the discovery of ATA as a potent inhibitor suggests that small organic molecules could serve as therapeutic drugs by blocking AβO binding. Remarkably, this compound has been tested previously in an Alzheimer’s mouse model, where it proved nontoxic, reached the brain after oral administration, and reversed memory impairment while showing no evidence of organ toxicity [57]. The authors reported this activity to be related to a mechanism involving the membrane attack complex. Our demonstration that ATA prohibits AβO binding in multiple assays provides an alternative mechanism, in which ATA could improve memory deficits in Alzheimer’s mice by blocking the binding of synaptotoxic Aβ oligomers.

## Materials and Methods

### Materials

Aβ_1–42_ and N-terminally biotinylated Aβ_1–42_ were purchased from American Peptide. Hamm’s F12 media was from Caisson. Palmitoyl-Oleoyl-Phosphatidylcholine (POPC) was from Avanti Polar Lipids. N-dodecyl-β-D-maltoside (DβM) was from Sigma Aldrich. Rat cerebral cortex was purchased from Pelfreez Biologicals. His-tag Isolation & Pulldown Dynabeads were from Invitrogen. Vectastain ABC reagent was from Vector Laboratories. AlphaLISA and AlphaScreen Ni-chelate Acceptor beads and AlphaScreen Streptavidin Donor beads were from Perkin Elmer. PrP-specific antibody (6D11) was from Signet Laboratories. NR1 and mGluR5 antibodies were from Santa Cruz Biotechnology. GluR2-specific antibody was from Chemicon. Antibodies to the insulin receptor α subunit were from Biosource. Phosphodetect insulin receptor pTyr_1162/1163_-specific antibody was from EMD Millipore. MSP1E3D1 is purified from E. coli as previously described [58] and stored lyophilized at −20°C until use. Oligomer-specific monoclonal antibodies NU4 and NU2 were produced as described [59]. Aurin tricarboxylic acid (CAS 4431-00-9), Epigallocatechin (CAS 970-74-1), Methylatropine nitrate (CAS 52-88-0), and Amphotericin B (CAS 1397-89-3) were from Sigma-Aldrich. Perseitol (CAS 527-06-0) was from Carbosynth Limited (Compton, Berkshire, UK). Spectrum collection compounds were from MicroSource Discovery Systems Inc., Gaylordsville, CT.

### Methods

Northwestern University's IACUC and Office of Laboratory Animal Welfare (A3283-01) approved the vertebrate animal protocol used in this work (Protocol 2012–983 v1). Briefly, timed-pregnant rats were euthanized by carbon dioxide inhalation and decapitation and embryos were anesthetized by inducing hypothermia prior to rapid decapitation.

### AβO preparation

AβOs were prepared as previously described [60,61]. Biotinylated AβOs used 1 part DMSO-solubilized biotinylated Aβ peptide to 5 parts unlabeled peptide. Ultrafiltration to separate high and low molecular weight AβOs was performed using Amicon Ultra 50K centrifugal concentrators (Millipore) according to the manufacturer’s instructions.

### Synaptic membrane isolation

Synaptic plasma membranes were isolated using an adaptation of published techniques [62]. Frozen rat cerebral cortex was thawed briefly in ice-cold PBS with 0.32 M sucrose and protease inhibitor cocktail (Roche). 3–4 grams were used per 24 mL sucrose solution. The cortex was homogenized with 12 strokes using a motor-driven glass/Teflon homogenizer. All centrifugation was carried out at 4°C using a Beckman SW-28 rotor. The homogenate was cleared at 800g for 20 minutes and the supernatant was spun at 9,000g for 20 minutes resulting in the crude synaptosomal pellet. The pellet was washed once using 0.32 M sucrose and re-pelleted. The synaptosomes were lysed by resuspending the pellet in ice-cold 20 mM Tris pH 7.5, manually homogenizing with a glass/Teflon homogenizer, and incubating on ice for 30 minutes. The lysed membranes were pelleted at 25,000g for 20 minutes. The pellet was thoroughly resuspended in 4 mL ice-cold H_2_O and mixed with 4 mL of 2.2 M sucrose. A sucrose gradient was assembled by layering 8 mL of 0.855 M sucrose and 8 mL of 0.32 M sucrose, and the samples were spun for 2.5 hours at 65,000g. The synaptic plasma membranes that accumulate at the interface between the 1.1 M and 0.855 M sucrose layers were collected, washed with PBS with protease inhibitor cocktail, and stored at—80°C. Protein concentration was measured using the BCA assay (Thermo Scientific).

Where used, synaptic plasma membranes were biotinylated by the addition of EZ Link sulfo-NHS-LC-LC-biotin (Pierce) to a concentration of 2 mM in PBS and incubation on ice for 90 minutes. The membranes were washed 3 times in PBS by centrifugation to remove residual unreacted biotinylating agent and resuspended to the original volume.

### Lipid extraction from synaptic membranes

Synaptic plasma membrane lipids were extracted using a protocol adapted from Breckenridge et al. [34]. Synaptic plasma membranes were suspended in methanol and then chloroform at a 2:1 C:M ratio. The final protein concentration during extraction was 0.1–0.15 mg mL^−1^. The precipitated protein was removed by centrifugation (20 minutes at 1,000g) and the pellet was washed with 1:2 C:M to extract remaining lipids. The supernatants from each centrifugation step were combined and adjusted to 2:1 C:M by adding extra chloroform and 0.2 volumes 100 mM KCl was added, resulting in a phase separation. The lower, lipidic phase was collected and dried to a film under N_2_. The lipid film was dissolved in chloroform and stored at—20°C. The concentration of phospholipids was assayed using the assay for total phosphorus [63].

### AβO binding to synaptosomes

Rat cortical synaptosomes were prepared according to Dodd et al. [64] and stored at—80°C until use. Synaptosomes were incubated with 0–1,000 nM AβOs in Hamm’s F12 with 0.1% (wt./vol) BSA for 1 hour at 37°C. Unbound AβOs were removed by two washes in PBS/BSA and one wash in PBS. Washes consisted of centrifugation at 10,000g for 5 minutes, resuspension in wash buffer, and a 10-minute incubation at 4°C with rotation. AβO-bound synaptosomes were spotted on nitrocellulose blotting membrane and the membrane was blocked with 5% (wt./vol) nonfat dry milk in TBS + 0.1% (vol/vol) Tween 20. The blot was probed using blocking buffer containing 1.5 μg mL^−1^ NU2, followed by HRP-linked anti-mouse IgG at a dilution of 1:20,000. *Modification for secondary drug screen* Active compounds identified in the HTS approach or an equivalent volume of DMSO were pre-incubated at a concentration of 10 μM with synaptosomes for 30 minutes at room temperature prior to the addition of 100 nM AβOs. Subsequent steps were identical to the above method.

### Immunoprecipitation and immunodepletion of AβO receptor complexes in synaptosomes

Synaptosomes were incubated for 1 hour at 37°C with 300 nM AβOs in F12. AβO-bound synaptosomes were washed 3 times in PBS and incubated 1 hour at 4°C with 2.5 μg mL^−1^ NU2 in PBS. Synaptosomes were washed 3 times in PBS and incubated in F12 with 0.2% (vol/vol) Triton and 0.1% (wt./vol) deoxycholate. Anti-mouse IgG Dynabeads were added to the detergent solution and rotated at 4°C overnight to immunoprecipitate AβO binding complexes. Eluates were applied to SDS-PAGE or dot blots. SDS-PAGE gels were silver stained. Dot blots were probed for NU2 and AβOs using HRP-linked anti-mouse IgG and M69/2 polyclonal AβO antibody [65], respectively.

### Preparation of SMPL Nanodiscs

To promote the formation of Nanodiscs containing single synaptic proteins, excess MSP and POPC were used to yield on average one membrane protein in five Nanodiscs. Chloroform-solubilized POPC (Avanti Polar Lipids) was dried under a stream of N_2_ to form an even film in a glass test tube and stored under vacuum for 4 hours to remove residual solvent. The dried POPC film was resuspended to a POPC concentration of 50 mM using 100 mM sodium cholate and alternately sonicated, vortexed, and incubated under warm running water until the solution becomes clear. MSP1E3D1 was added to a MSP1E3D1:POPC ratio of 1:130 and the solution was incubated at 4°C for 2 hours with gentle agitation. Membranes were solubilized by pelleting and resuspending in 1% (wt./vol) n-dodecyl-β-d-maltoside (DβM) to a protein concentration of 2 mg mL^−1^ and incubating at 4°C for 2 hours on a rotator. Solubilized membranes were added to the MSP1E3D1/POPC mixture and the final volume was adjusted with 50 mM Tris pH 7.5, 150 mM NaCl, 0.5 mM EDTA to result in a final POPC between 5 and 20 mM and a sodium cholate concentration between 12–40 mM. This mixture was incubated at 4°C for 2 hours with gentle agitation. Nanodisc self-assembly was initiated by overnight detergent removal using H_2_O-equilibrated Amberlite XAD-2 detergent removal beads, which were drained and added to the Nanodisc reaction until they account for one half the final volume. Reactions were rotated to keep the detergent removal beads in suspension. Detergent removal beads were filtered out and the nascent Nanodiscs were purified using Ni-NTA agarose resin (Qiagen). Dialysis against at least 3000 volumes PBS was used to remove imidazole, which interferes with His-tag immobilization experiments. Total protein concentration was measured using the BCA assay. Nanodiscs were flash frozen in liquid N_2_ and stored at—80°C until use.

### Size Exclusion Chromatography

Size exclusion chromatography was carried out on an AKTAexplorer 10 (GE Life Sciences) at a flow rate of 0.5 mL min^−1^ using a Superdex200 10/300 GL column. Data was analyzed using Unicorn 5.11 (GE Healthcare).

### Glutamate binding assay

25 mm Whatman GF/B filters were soaked in 0.3% (wt./vol) polyehtylenimine for 1 hour at room temperature. Nanodiscs were incubated at 0.5 mg mL^−1^ with 10 μM ^3^H glutamate (1 nCi μL^−1^) with or without 1 mM cold glutamate for 1 hour at room temperature. Filters were dried under vacuum. The reactions were applied directly to the filters and washed with 50 mM Tris pH 7.5, 150 mM NaCl, 0.5 mM EDTA. Bound ^3^H glutamate was quantified using a Beckman Coulter LS6500 scintillation counter.

### Tyrosine kinase activity assay in SMPL Nanodiscs

SMPL Nanodiscs were immobilized on His-tag Isolation and Pulldown Dynabeads and exposed to 60 units of alkaline phosphatase in 50 mM HEPES pH 7.5 + 0.1% (wt./vol) BSA for 1 hour at 4°C. The immobilized Nanodiscs were incubated in a buffer containing 100 ng mL^−1^ BDNF, 500 ng mL^−1^ NGF, 1 μg mL^−1^ EGF, 1.7 μM insulin, 10 mM MgCl_2_, 2 mM MnCl_2_, 5 mM NaF, and 100 μM activated NaVO_4_ for 1 hour at room temperature. The beads were washed in HEPES buffer and eluted in Laemmli buffer with 50 mM DTT and 250 mM imidazole. The eluates were resolved by SDS-PAGE and transferred to nitrocellulose. Blots were probed using Biosource phosphotyrosine-specific antibody (1:1000) or EMD 407707 IR_β_pTyr_1162/1163_ antibody (1:1000).

### Magnetic bead-based ELISA assay for AβO binding

All binding and washing steps were carried out in PBS with 0.1% (wt./vol) BSA and 25 mM imidazole. SMPL Nanodiscs or control Nanodiscs (20 μg) were immobilized on His-tag Isolation and Pulldown Dynabeads (20 μL) for 30 minutes. The beads were washed 3 x 10 minutes at 4°C. The bead-immobilized Nanodiscs were exposed to 100 nM biotinylated AβOs for 1 hour at 37°C. Following 3 x 10-minute washes, bound biotinylated AβOs were detected for 1 hour at 4°C using Vectastain (reagents A and B were pre-complexed for 30 minutes at 1:1,000 before use). The magnetic beads were washed 3 x 10 minutes and incubated at room temperature in 750 μL colorimetric HRP substrate (BioRad) or Femto Elisa Substrate (G-Biosciences) until color develops. The Dynabeads were immobilized and the supernatants removed to stop the reaction. The absorbance at 405 nm (BioRad) or 650 nm (G-biosciences) was measured using duplicate 200 μL samples on a Dynex plate reader. The curve fitting is performed using the “One site—Total and nonspecific binding” preset in Graphpad Prism having the form: Y = B_max_*X/(Kd+X) + N.S.*X + Background where N.S. is the nonspecific binding signal. The fitting constants were used to generate a continuous curve for the specific (Y = B_max_*X/(Kd+X)) binding component.

### PrPC removal from Nanodiscs and Neurons

PrP^C^ removal from SMPL Nanodiscs was performed by treatment with PIPLC (Sigma-Aldrich) as a part of the AβO binding assay described above. SMPL Nanodiscs were immobilized on His-tag Isolation and Pulldown Dynabeads and split into four equivalent microcentrifuge tubes. Immobilized Nanodiscs were exposed to 0, 0.05, 0.1, or 0.2 units mL^−1^ PIPLC for 2 hours at room temperature before their exposure to 100 nM AβOs. After exposure to HRP substrate, beads were washed and eluted with 1X Laemmli buffer for SDS-PAGE/Western blot analysis using the 6D11 antibody against PrP^C^.

PrP^C^ was removed from cultured neurons by a 1-hour pre-treatment with 0.2 units mL^−1^ PIPLC at 37°C. Without washing, fluorescent AβOs (100 nM) were added to the culture media and incubated at 37°C for 30 minutes. Following 3 washes in Neurobasal media (Life Technologies), cells were fixed using 3.7% (wt./vol) formaldehyde for 5 minutes at a 1:1 dilution followed by 5 minutes at the undiluted concentration and counterstained for PrP^C^ using 6D11 antibody (Signet) at 1:1000 and anti-mouse Alexa Fluor-633 secondary antibody at 1:1000. Fluorescence images were captured at 60X using a Nikon Eclipse TC2000-U microscope with a Photometrics CoolSNAP HQ CCD camera and analyzed using ImageJ (http://rsbweb.nih.gov/ij/).

### Affinity precipitation from synaptosomes and SMPL Nanodiscs

Dynabeads displaying anti-mouse IgG (Invitrogen) were complexed to NU2 by incubating overnight at 4°C in 4 μg NU2 in PBS/BSA per 100 μL Dynabeads. The beads were washed 3 x 10 minutes in PBS with 0.1% (wt./vol) BSA, then 1 mL of 300 nM AβOs in Hamm’s F12 with 0.1% (wt./vol) BSA are added. Reactions were rotated 5 hours at 4°C. The magnetic beads were washed three times in F12/BSA. Nanodiscs at 200 μg mL^−1^ in F12/BSA were added to the beads and rotated overnight at 4°C to allow the affinity precipitation of Nanodiscs containing AβO binding proteins. The beads were washed three times with 1 mL of PBS/BSA to remove non-interacting Nanodiscs. All residual buffer was removed by centrifugation and the magnetic pellets were pre-eluted using 20 μL of 80mM cholate to disrupt the Nanodiscs and release MSP1E3D1 and nonspecific proteins not associating with immobilized AβOs. The pre-eluted beads were washed in PBS and the remaining proteins were eluted with 20 μL of 0.2% (wt./vol) SDS for 10 minutes at room temperature, vortexing occasionally to keep the beads in suspension. The eluates were analyzed by SDS PAGE followed by silver staining or Western blotting using Vectastain (if biotinylated SMPL Nanodiscs were used).

### AlphaScreen assays

In the testing phase (Fig 5) Robotic liquid handlers (Biomek Span-8 or AP96) were used to apply 10 μL each of 1 μg mL^−1^ Nanodiscs and 200 nM biotinylated AβOs—both diluted in PBS with 0.1% (wt./vol) BSA—into the wells of a 384-well plate (Grenier). When applicable, NU2 antibody was applied using a Labcyte Echo 550 acoustic liquid handler before adding AβOs. The plate was incubated at room temperature on a plate shaker for 1 hour and AlphaLISA nickel-chelate acceptor beads were added to each well using the Labcyte Echo 550 resulting in a final concentration of 1 μg mL^−1^. After one hour of co-incubation at room temperature on a plate shaker, 1 μg mL^−1^ of AlphaScreen streptavidin donor beads were added using the Labcyte Echo 550. The final reaction was incubated one hour at room temperature on a plate shaker. The luminescence signal was read using a Perkin-Elmer EnSpire plate reader set to excite at 680 nm for 180 ms and collect at 570 nm for 550 ms.

In the screening phase (Fig 6B) Nanodiscs (POPC or SMPL) diluted to 20 μg mL^−1^ in filtered PBS with 0.1% BSA and briefly centrifuged. In subdued lighting, nickel-chelate acceptor beads were added to a concentration of 20 μg mL^−1^ and mixed thoroughly by inversion. Tubes were briefly centrifuged and incubated for 1 hour at RT on a rotator covered with foil to block light. Compounds (10 mM stock in DMSO at -20°C) were thawed and centrifuged. An Echo550 acoustic liquid handler was used to transfer 80 nL into duplicate wells of an opaque white 384-well assay plate. The Nanodisc/acceptor bead complex is transferred to a conical-bottomed 96-well plate (45 μL per well) and a Biomek AP96 pipettor is used to transfer 10 μL to each well of the assay plate containing the compounds in DMSO. The plate is sealed with adhesive foil and incubated for 1h at RT on a plate shaker at 800 rpm. Biotinylated AβOs flash frozen and stored at -80°C as single-use aliquots were diluted to 400 nM in PBS with 0.1% BSA, transferred to a 96 well-plate (25 μL per well) and to the assay plate in 5 μL per well to give a final concentration of 100 nM AβOs. The plate is sealed and incubated for 1 hour on the plate shaker as before. In subdued lighting, AlphaScreen streptavidin donor beads were diluted to 80 μg mL^-1^ in PBS with 0.1% BSA and transferred to the assay plate as described for the AβOs in 5 μL aliquots per well. The assay plates were incubated 1h at room temperature on a plate shaker and luminescence signal was read on an EnSpire plate reader as before.

In the biotinylated hexahistidine counterscreening assay, biotinylated hexahistidine (Perkin-Elmer) was diluted to 10 nM in PBS with 0.1% BSA and AlphaScreen nickel-chelate acceptor beads were added to a concentration of 40 μg mL^-1^ and incubated at room temperature for 1 hour. Compounds were transferred using an Echo550 acoustic liquid handler to an opaque white 384-well plate to give concentrations of 50, 25, and 10 μM in 20 μL. 10μL His^6^-acceptor bead complex was transferred to each well using a Biomek AP96, followed by 10 μL of streptavidin donor beads at 40 μg mL^-1^ in PBS with 0.1% BSA. The plate was incubated 1 hour at room temperature and read with an EnSpire plate reader using the same settings as the primary assay plates.

In the counterscreening assay testing for Nanodisc-disrupting compounds, Nanodiscs containing biotinylated synaptic membranes were prepared as above. Nanodiscs were diluted to 1 μg mL^-1^ in PBS with 0.1% BSA and incubated for 1 hour at room temperature with nickel-chelate AlphaScreen acceptor beads at a concentration of 20 μg mL^-1^. Streptavidin donor beads were added to the tubes to a concentration of 20 μg mL^-1^ and incubated 1 hour at room temperature. Compounds were transferred to a 384-well plate using the Echo550 acoustic liquid handler to give final concentrations of 10, 25, and 50 μM in 20 μL. Sodium cholate was used as a positive control, added separately to 20 mM. The biotinylated SMPL/donor/acceptor complex was transferred to the plate using a Biomek AP96 and the plate was incubated on a plate shaker for 1 hour at room temperature. The plate was read on an EnSpire plate reader using the same settings as the primary assay plates.

### Hippocampal neuron culture and effect of STI and screening compounds on AβO binding

Hippocampal nerve cell cultures were prepared on glass cover slips using embryonic day 18 rats according to published procedures [66]. Bovine soybean trypsin inhibitor type II (Sigma Aldrich) was reconstituted in PBS to a concentration of 20 mg mL^-1^ and biotinylated using a 5:1 ratio of EZ Link NHS-lc-biotin (Pierce) to protein for 30 minutes at room temperature. Small molecule inhibitors were dissolved in DMSO at a stock concentration of 10 mM. Biotinylated STI or small molecule inhibitors were added to the cultures for 30 minutes at 37°C, followed by the addition of AβOs to a concentration of 100 nM. The AβOs were allowed to incubate for 30 minutes at 37°C and the cells were washed three times with 1 mL Neurobasal media at 37°C. To fix, 1 mL 3.7% formaldehyde at 37°C was added to the final wash and the cells were incubated at room temperature for 5 minutes before replacing with undiluted 3.7% formaldehyde for an additional 5 minutes. Fixed cells were washed 4x10 min with PBS and blocked for 1 hour using PBS with 10% normal goat serum (Gibco) and 0.1% Triton X100. Primary antibody solution was prepared in blocking buffer and contained 1.5 μg mL^-1^ NU2 and 1:1000 anti-TUBB3 (Protein Tech). Coverslips were incubated in primary antibody solution overnight at 4°C and washed 3x10 min with PBS. Secondary antibody solution was prepared in 10% blocking buffer. For the STI experiment the secondary antibodies were used at 1:1000 dilutions and were Alexa Fluor-647 goat anti-rabbit IgG, Alexa Fluor-488 goat anti-mouse IgG, and Streptavidin Alexa Fluor-555 conjugate. For the small molecule inhibitors experiments the secondary antibodies were used at 1:1000 dilutions and were Alexa Fluor-488 goat anti-rabbit IgG and Alexa Fluor-555 goat anti-mouse IgG. Coverslips were mounted to glass microscope slides using ProLong Gold Antifade Reagent with DAPI (Molecular Probes). Fluorescence images were captured as above, using tubulin signal and not AβOs to select neurons for imaging. Bound AβOs were quantified as pixel intensity per unit branch length using custom macros in Cell Profiler as described [67].

## Supporting Information

S1 Fig Glutamate binding to synaptic plasma membrane and effect of trypsin treatment on Glutamate binding to SMPL Nanodiscs. ^3^H glutamate binding to synaptic plasma membranes was assessed in the absence and presence of a 100-fold excess of cold glutamate (a). ^3^H glutamate binding to SMPL Nanodiscs assembled to contain trypsin-treated synaptic plasma membranes was assessed compared to Nanodiscs containing intact membranes (b). CPM—counts per minute.
(TIF)

S2 Fig Soybean trypsin inhibitor blocks AβO binding to Neurons and synaptic SMPL Nanodiscs. Representative images from immunocytochemistry experiments (a) show that AβO binding (green immunofluorescence) is greatly reduced after a 30-minute pre-treatment with STI (right panel). Red immunofluorescence denotes binding of STI to neurons as identified by biotin detection. STI treatment reduces AβO binding to SMPL Nanodiscs in an AlphaScreen assay (b), reducing the signal by 67% relative to POPC Nanodiscs. Quantification of AβO binding to neurons reveals an 87% reduction of neuronal binding in the presence of STI (c).
(TIF)

S3 Fig AlphaScreen assay well-to-well correlation and z scores. (a) A correlation plot comparing duplicate wells containing the same components of the Spectrum Collection analyzed in the primary AlphaScreen Assay shows an R^2^ value of 0.78. (b) A histogram of z factors calculated using internal POPC and SMPL standards on each of 17 assay plates in the primary AlphaScreen assay. The average z factor was 0.59 and ranged from 0.4 to 0.805.
(TIF)
