# Supplementary figures and images for: Nanoscale Synaptic Membrane Mimetic Allows Unbiased High Throughput Screen That Targets Binding Sites for Alzheimer’s-Associated Aβ Oligomers
Source: PLoS One. 2015 Apr 30;10(4):e0125263. doi: 10.1371/journal.pone.0125263 (PMC4415972; doi:10.1371/journal.pone.0125263)

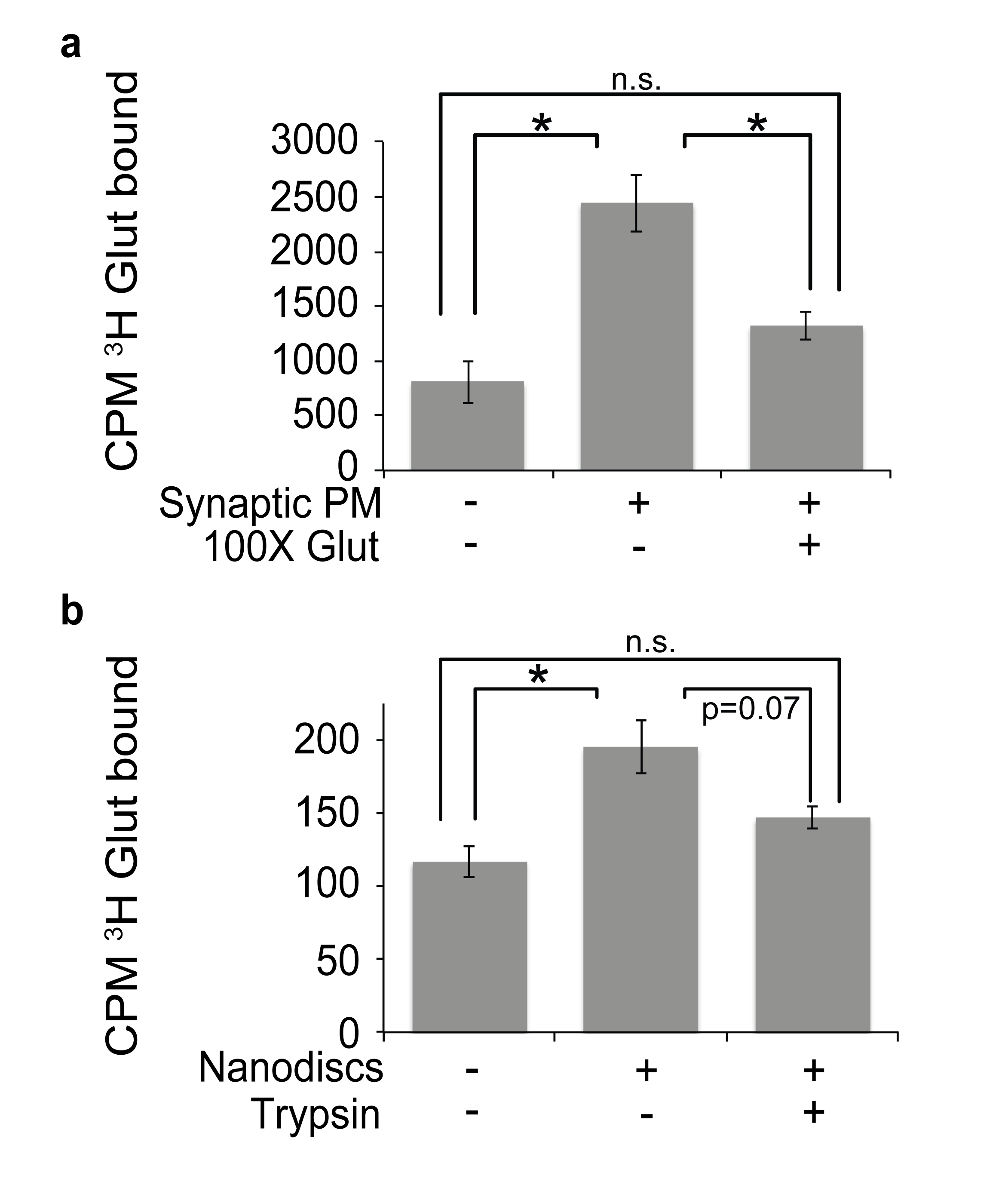

Supplement: S1 Fig — 3H glutamate binding to synaptic plasma membranes was assessed in the absence and presence of a 100-fold excess of cold glutamate (a). 3H glutamate binding to SMPL Nanodiscs assembled to contain trypsin-treated synaptic plasma membranes was assessed compared to Nanodiscs containing intact membranes (b). CPM—counts per minute. (TIF) [file pone.0125263.s001.tif]

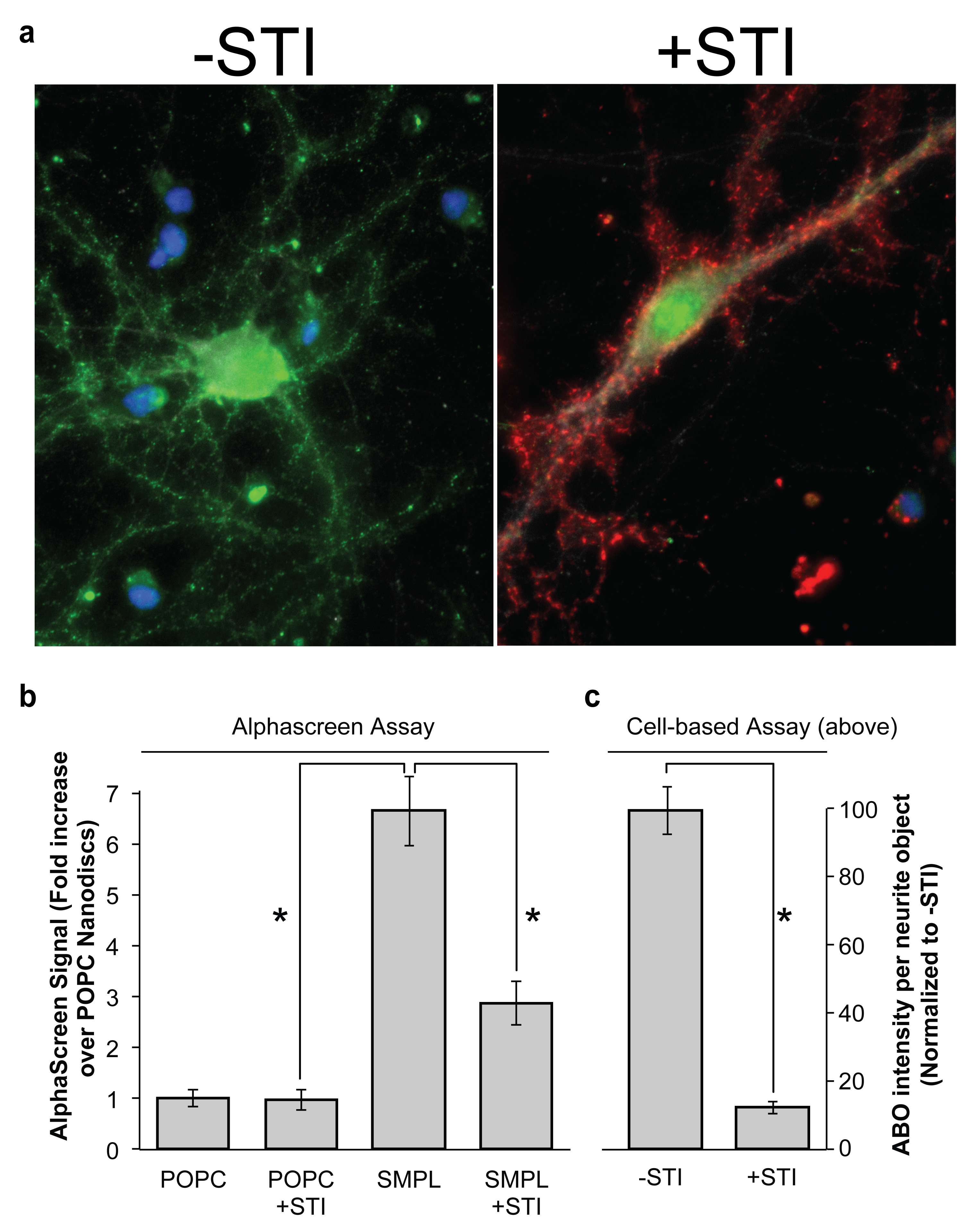

Supplement: S2 Fig — Representative images from immunocytochemistry experiments (a) show that AβO binding (green immunofluorescence) is greatly reduced after a 30-minute pre-treatment with STI (right panel). Red immunofluorescence denotes binding of STI to neurons as identified by biotin detection. STI treatment reduces AβO binding to SMPL Nanodiscs in an AlphaScreen assay (b), reducing the signal by 67% relative to POPC Nanodiscs. Quantification of AβO binding to neurons reveals an 87% reduction of neuronal binding in the presence of STI (c). (TIF) [file pone.0125263.s002.tif]

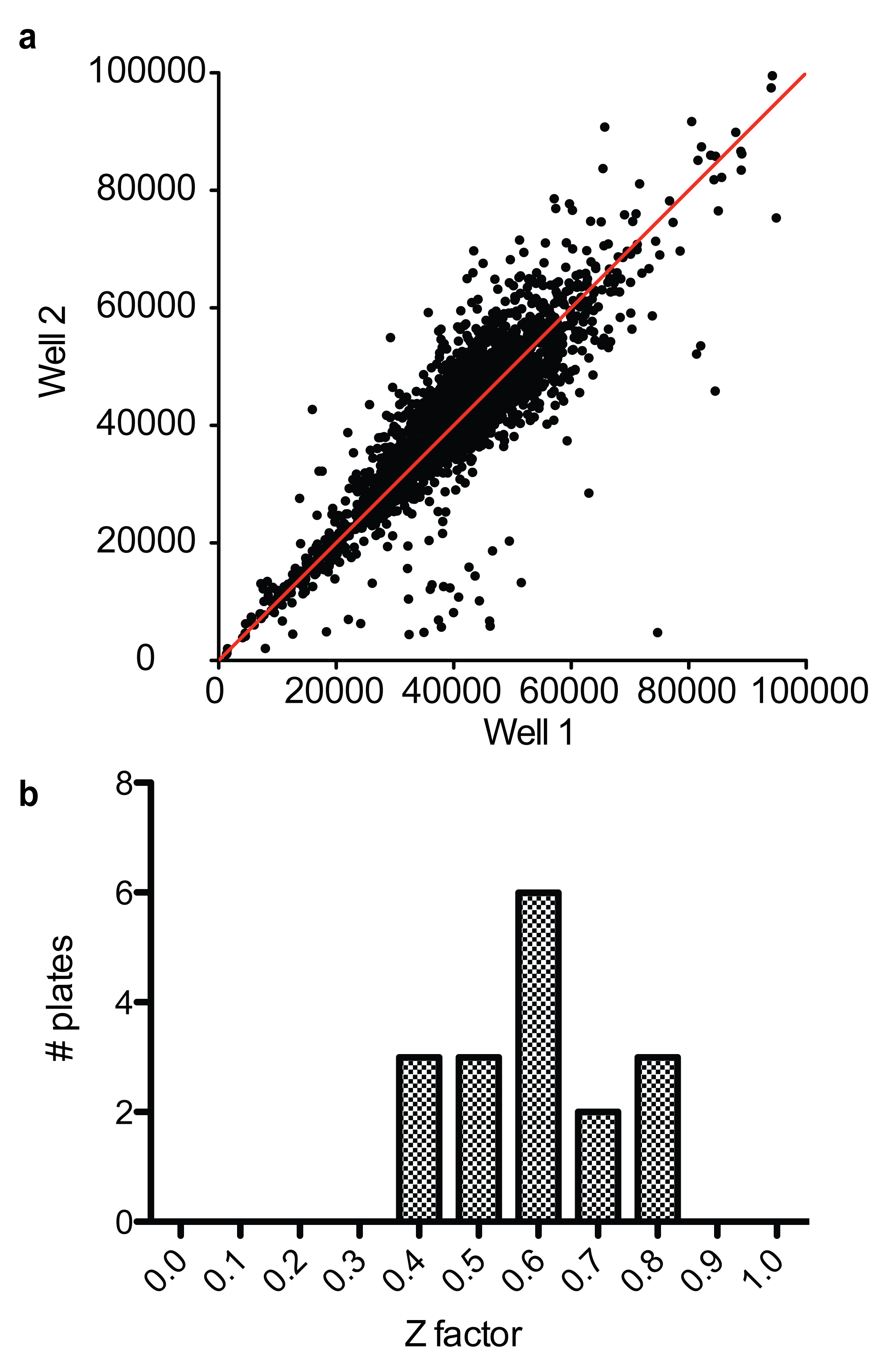

Supplement: S3 Fig — (a) A correlation plot comparing duplicate wells containing the same components of the Spectrum Collection analyzed in the primary AlphaScreen Assay shows an R2 value of 0.78. (b) A histogram of z factors calculated using internal POPC and SMPL standards on each of 17 assay plates in the primary AlphaScreen assay. The average z factor was 0.59 and ranged from 0.4 to 0.805. (TIF) [file pone.0125263.s003.tif]
